# Erythropoietin Inhibits Gluconeogenesis and Inflammation in the Liver and Improves Glucose Intolerance in High-Fat Diet-Fed Mice

**DOI:** 10.1371/journal.pone.0053557

**Published:** 2013-01-10

**Authors:** Ran Meng, Dalong Zhu, Yan Bi, Donghui Yang, Yaping Wang

**Affiliations:** 1 Department of Endocrinology, Nanjing Drum Tower Hospital, Nanjing University School of Medicine, Nanjing, China; 2 Department of Medical Genetics, Nanjing University School of Medicine, Nanjing, China; 3 Jiangsu Key Laboratory of Molecular Medicine, Nanjing University, Nanjing, China; Pennington Biomedical Research Center, United States of America

## Abstract

Erythropoietin (EPO) has multiple biological functions, including the modulation of glucose metabolism. However, the mechanisms underlying the action of EPO are still obscure. This study is aimed at investigating the potential mechanisms by which EPO improves glucose tolerance in an animal model of type 2 diabetes. Male C57BL/6 mice were fed with high-fat diet (HFD) for 12 weeks and then treated with EPO (HFD-EPO) or vehicle saline (HFD-Con) for two week. The levels of fasting blood glucose, serum insulin and glucose tolerance were measured and the relative levels of insulin-related phosphatidylinositol 3-kinase (PI3K)/Akt, insulin receptor (IR) and IR substrate 1 (IRS1) phosphorylation were determined. The levels of phosphoenolpyruvate carboxykinase (PEPCK), glucose-6- phosphatase (G6Pase), toll like receptor 4 (TLR4), tumor necrosis factor (TNF)-α and IL-6 expression and nuclear factor-κB (NF-κB) and c-Jun N-terminal kinase (JNK), extracellular-signal-regulated kinase (ERK) and p38 MAPK activation in the liver were examined. EPO treatment significantly reduced the body weights and the levels of fasting blood glucose and serum insulin and improved the HFD-induced glucose intolerance in mice. EPO treatment significantly enhanced the levels of Akt, but not IR and IRS1, phosphorylation, accompanied by inhibiting the PEPCK and G6Pase expression in the liver. Furthermore, EPO treatment mitigated the HFD-induced inflammatory TNF-α and IL-6 production, TLR4 expression, NF-κB and JNK, but not ERK and p38 MAPK, phosphorylation in the liver. Therefore, our data indicated that EPO treatment improved glucose intolerance by inhibiting gluconeogenesis and inflammation in the livers of HFD-fed mice.

## Introduction

Excessive caloric intake usually causes glucose intolerance and insulin resistance, the hallmarks of metabolic syndrome and type 2 diabetes (T2D), which are important health challenges in the world [1], [2]. Currently, there are many medicines available and under the development for the treatment of glucose intolerance and insulin resistance. However, the therapeutic efficacy of these medicines and their safety profiles remain questionable. Therefore, the discovery and development of new medicines will be of great significance. Theoretically, glucose homeostasis is regulated by the balance of gluconeogenesis, glycogenesis, glyogenolysis and glucose metabolism. The gluconeogenesis is a critical process to convert non-carbohydrate carbon substrates, such as pyruvate, lactate, glycerol, and glucogenic amino acids, to glucose, regulating glucose metabolism in humans. Thus, inhibition of gluconeogenesis may be ideal for the improvement of glucose intolerance and insulin resistance, and a promising strategy for the treatment of metabolic syndrome and T2D.

Erythropoietin (EPO), a hematopoietic growth factor, is predominantly produced in the kidney, and has been widely used in patients with anemia from renal diseases and myelodysplasia following chemotherapy or radiotherapy [3]. Interestingly, treatment with EPO has been shown to ameliorate insulin resistance in patients, who have end-stage renal disease (ESRD) and undergo hemodialysis therapy [4], [5]. Furthermore, EPO transgenic mice display significantly lower levels of blood glucose, insulin and HA1C, and are resistant to high fat diet (HFD)-induced glucose intolerance and insulin resistance [6], [7]. In contrast, EPO receptor (EPOR) null mice develop insulin resistant [8]. Accordingly, EPO may regulate glucose tolerance and insulin sensitivity. However, how EPO regulates glucose metabolism and whether EPO regulates gluconeogenesis have not been explored.

Insulin plays a crucial role in the regulation of glucose metabolism [9]. Insulin can bind to insulin receptor (IR) and activate IR tyrosine kinase, which activates IR substrate (IRS). Subsequently, the activated IRS recruits and activates the phosphatidylinositol 3-kinase (PI3K) and Akt, leading to glucose transportation, glucagon synthesis, and inhibiting gluconeogenesis in the liver [10]–[12]. Impairment in the PI3K/Akt pathway is associated with glucose intolerance and insulin resistance as well as increased expression of phosphoenolpyruvate carboxykinase (PEPCK) and glucose-6-phosphatase (G6Pase), two rate-limiting enzymes for hepatic glucose production [13], [14]. Hence, enhancement of the PI3K/Akt activation may be a promising strategy for improving glucose tolerance and insulin sensitivity. Interestingly, EPO can activate, through the EPOR, the PI3K/Akt pathway in many organs [15]–[18]. Given that the EPOR is expressed in the liver, we hypothesize that EPO may activate the PI3K/Akt pathway to inhibit the PEPCK and G6Pase expression and gluconeogenesis in the liver, improving glucose tolerance and insulin sensitivity, even in obese animals.

Chronic low-grade inflammation contributes to the development of obesity-related glucose intolerance and insulin resistance [19]. The toll like receptor 4 (TLR4) recognizes endogenous free fat acid and exogenous pathogens [20], [21] and engagement of TLR4 activates the nuclear factor κB (NF-κB) [22] and mitogen-activated protein kinase (MAPK) subfamily members [23], including the extracellular-signal-regulated kinase (ERK), c-Jun N-terminal kinases (JNK) and p38 MAPK, as well ascytokines [24], and stimulates the production of inflammatory cytokines, such as tumor necrosis factor (TNF)-α and interleukin (IL)-6, contributing to glucose intolerance and insulin resistance. Given that EPO treatment inhibits the NF-κB, ERK and JNK activation, and TNF-α and IL-6 production in animal models of hepatic injury [25]–[28], we hypothesize that EPO may also inhibit the HFD-induced chronic inflammation, contributing to the improvement of glucose intolerance and insulin resistance.

In the present study, we fed C57BL/6 mice with HFD to establish glucose intolerance and investigated the effects of EPO treatment on glucose tolerance and the potential molecular mechanisms underlying the action of EPO in regulating gluconeogenesis and inflammation in the liver.

## Materials and Methods

### Animals and experimental procedures

All experimental procedures were approved by the Animal Care and Use Committee of Nanjing University (Approval ID: 2008000027). Male C57BL/6 mice at 4 weeks of age were purchased from the Animal Center of Yangzhou University (Yangzhou, Jiangsu, China) and housed in a specific pathogen-free facility throughout the experimental period. The mice were fed with HFD (60% kcal fat, 20% kcal carbohydrates, 20% kcal protein, Guangzhou Animal Experiment Center, Guangzhou, China), or with normal chow diet (10% kcal fat, 70% kcal carbohydrates, 20% kcal protein) for 12 weeks. The HFD-fed mice were randomized and treated intraperitoneally with 1000 IU/kg recombinant human EPO (Sunshine Pharmaceutical, Shenyang, China) every other day for two weeks (HFD-EPO group) or with saline (HFD-Con group), respectively. The healthy control mice with normal chow diet were injected with saline (NC group). Their dietary intake and body weights were measured every other day. At the end of the experiment, their fasting blood samples were collected and the mice were sacrificed. Their liver tissues were harvested, weighed, snap frozen in liquid nitrogen, and stored at −80°C until use.

### ELISA

The blood samples of individual mice were centrifuged at 1,500 ×*g* for 10 min for preparing plasma or serum samples. The concentrations of plasma TNF-α and IL-6 were measured using the cytokine-specific ELISA kits, according to the manufacturers' instruction (RayBiotech, Norcross, USA). Similarly, the concentrations of serum insulin in individual mice were determined using the mouse insulin ELISA kit, according to the manufacturer's instructions (Linco, Charles, USA). The detection limitation for TNF-α, IL-6 and insulin was 50 pg/ml, 2 pg/ml and 0.2 ng/ml, respectively.

### Glucose tolerance test

After treatment with EPO or vehicle for two weeks, the mice were fasted for 6 h and injected intraperitoneally with glucose (1 g/kg body weight). The levels of tail venous blood glucose in individual mice were measured at 0, 30, 60 and 120 min post glucose challenge using test strips on an One Touch profile glucose meter (Johnson & Johnson, New Brunswick, USA). The area under the curve (AUC) for the levels of blood glucose over the experimental period was calculated.

### Western blot analysis

The liver tissue samples were obtained from individual mice and lyzed in NP40 lysis buffer (140 mM NaCl, 10 mM Tris (pH 7.4), 1 mM CaCl2, 1 mM MgCl2, 10% glycerol, 1% Nonidet P-40, 1 mM dithiothreitol, 0.5 mM phenylmethylsulfonyl fluoride, 2 ng/µl of aprotinin, 10 ng/µl of leupeptin), followed by centrifugation. After quantification of protein concentrations, the liver lysates (20 µg/lane) were separated by SDS-PAGE on 10% polyacrylamide gels, and electrotransferred onto polyvinylidene difluoride (PVDF) membranes (Millipore, Billerica, USA). The membranes were blocked with 5% fat-free milk and incubated with anti-IR, anti-IRS1 (Millipore, Billerica, USA), anti-JNK, anti-p-JNK (Thr183/Tyr185), anti-p-IRS1 (Ser307), anti-p-IR (Tyr1346), anti-Akt, anti-p-Akt (Ser473), anti-p38, anti-p-p38 (Thr180/Tyr182), anti-TLR4, anti-PEPCK, anti-G6Pase (Cell Signaling Technology, Danvers, USA), anti-GAPDH, anti-tubulin (Boster, Wuhan, China), respectively. The bound antibodies were detected using horseradish peroxidase (HRP)-conjugated anti-rabbit antibodies and visualized using enhanced chemiluminescence (ECL, Boster). The relative levels of target proteins to controls were determined by densimetric analysis using ImageJ software.

### RT-PCR and quantitative PCR analysis

Total RNA was extracted from the liver samples of individual mice by Trizol (Takara Bio, Shiga, Japan) and reversely transcribed into cDNA using M-MLV reverse transcriptase (Toyobo, Osaka, Japan). The relative levels of EPO receptor mRNA transcription in individual liver samples was determined by RT-PCR using the specific primers of 5′-CTA TGG CTG TTG CAA CGC GA-3′ (forward) and 5′-CCGAGG GCA CAG GAG CTT AG-3′ (reverse). The kidney tissue samples from the same mice were used as positive controls for testing the EPO receptor expression. The amplification was performed at 94°C for 2 min and subjected to 30 cycles of 94°C for 30 seconds, 62°C for 30 seconds, and 72°C for 50 seconds, followed by a final extension at 72°C for 7 minutes. The PCR products were analyzed by 1% agarose gel electrophoresis and imaged.

In addition, the relative levels of target gene mRNA transcripts to β-actin in the liver tissues of individual mice were determined by quantitative real-time PCR using the SYBR Premix Ex Taq (Takara Bio, Shiga, Japan) and specific primers on a StepOne Real-Time PCR system, according to the manufacturer's instructions (Applied Biosystems, Foster City, USA). The sequences of primers are listed in Table 1. The relative levels of the target gene mRNA transcripts to the β-actin were calculated by 2^−ΔΔCt^.

**Table 1:** The sequences of the primers.

Gene	Forward Primer	Reverse Primer
IL-6	5′-TCCAGTTGCCTTCTTGGGAC-3′	5′-GTGTAATTAAGCCTCCGACTTG-3′
TNF-α	5′-CAGGAGGGAGAACAGAAACTCCA-3′	5′-CCTGGTTGGCTGCTTGCTT-3′
PEPCK	5′-GAACTGACAGACTCGCCCTATGT-3′	5′-GTTGCAGGCCCAGTTGTTG-3′
G6Pase	5′-GTGCAGCTGAACGTCTGTCTGT-3′	5′-TCCGGAGGCTGGCATTGT-3′
TLR4	5′-AGAAAATGCCAGGATGATGC-3′	5′-ATTTTGTCTCCACAGCCACC-3′
β-actin	5′-CATCCGTAAAGACCTCTATGCCAAC-3′	5′-ATGGAGCCACCGATCCACA-3′

### The DNA-binding based ELISA for the measurement of NF-κB activity

Nuclear proteins were extracted from the liver tissue samples of individual mice using the nuclear protein extract kit, according to the manufacturers' instruction (Active Motif, Carlsbad, USA). The concentrations of proteins were quantified using a Bradford assay, and the activities of NF-κB p65 binding to the specific DNA oligonucleotides in individual samples were determined using the TransAM NF-κB p65 kit (Active Motif), as described previously [29], [30].

### Statistical analysis

Data are expressed as the means ± S.E.M. The different among groups was analyzed by one-way ANOVA and the least significant difference (LSD) using the SPSS 13.0 Program. A *P* value of <0.05 was considered statistically significant.

## Results

### Effects of EPO on body weights, food intakes, fasting blood glucose, fasting serum insulin and glucose tolerance in the HFD-fed mice

**Figure 1:**
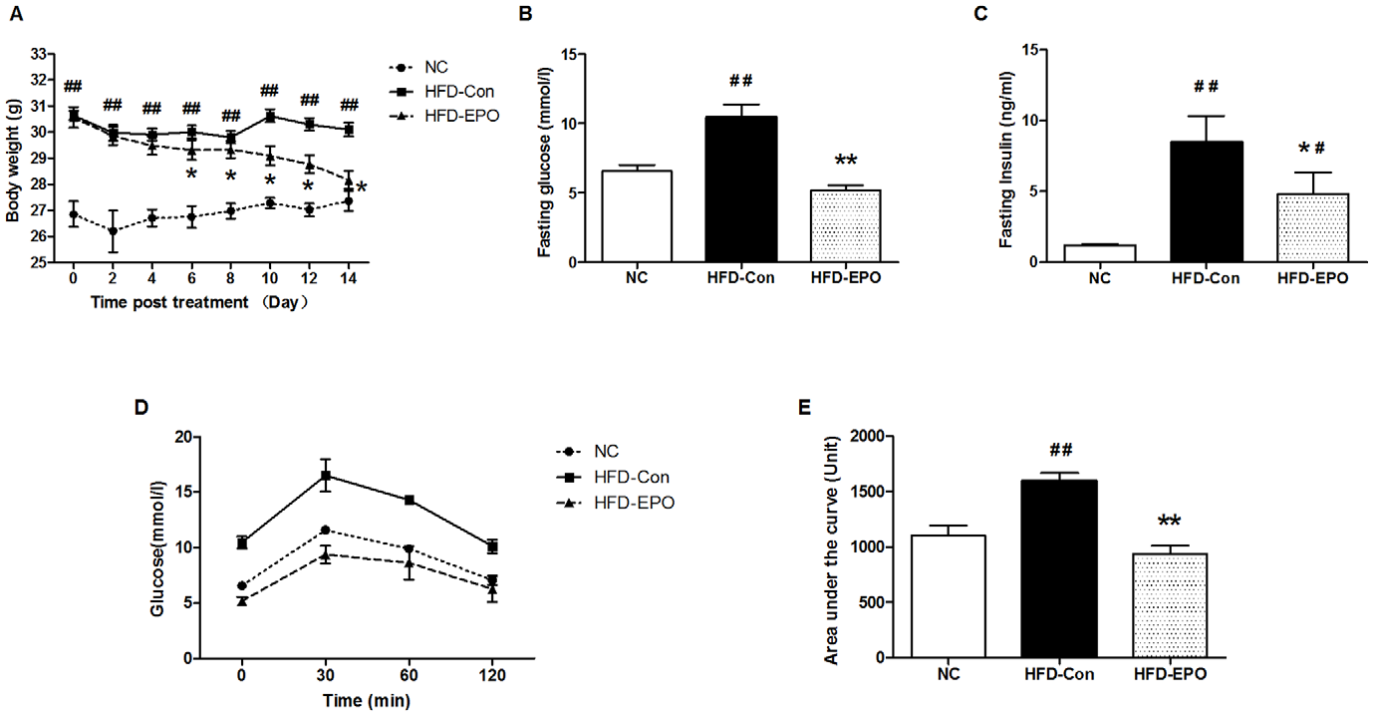
The effect of EPO on HFD-fed mice. C57BL/6 mice were fed with HFD for 12 weeks and treated with EPO (HFD-EPO) or injected with saline (HFD-Con) for two weeks. A control group (NC) of mice was fed with normal chow and injected with saline. The body weights and amounts of food consumed in individual mice were measured at the indicated time points. At the end of treatment, the mice were fasted and subjected to measurements of the levels of fasting blood glucose, fasting serum insulin and IPGTT. The AUC for blood glucose in individual mice was calculated. Data are present as the mean ± SEM of each group (n = 8) of mice from two-three separate experiments. (**A**) The body weights. (**B**) The levels of fasting blood glucose. (**C**) The levels of fasting serum insulin. (**D**) The glucose tolerance. (**E**) The values of AUC. There was no significant difference in the amounts of food intake among these groups of mice (data not shown). ^#^P<0.05 or ^##^P<0.01 vs. the NC group, *P<0.05 or **P<0.01 vs. the HFD-Con group.

To test the effect of EPO on glucose tolerance, C57BL/6 mice were fed with HFD or normal chow (NC group) for 12 weeks. The HFD-fed mice were randomly treated with EPO (HFD-EPO group) or vehicle saline (HFD-Con group) every other day for two weeks and the body weights in the different groups of mice were monitored every other day through the experimental period. The body weights in the HFD-EPO and HFD-Con groups of mice were comparable within 4 days post treatment and were significantly greater than that of the NC group of mice (p<0.05, Fig. 1A). Subsequently, the body weights in the HFD-EPO group of mice gradually decreased and were significantly less than that of the HFD-Con group (p<0.05). At two weeks post treatment, the body weights in the HFD-EPO group of mice were similar to that in the NC group (p>0.05). There was no statistically significant difference in the amounts of food intakes among the different groups of mice throughout the experimental period (data not shown).

Analysis of fasting blood glucose and fasting serum insulin indicated that the concentrations of fasting blood glucose in the HFD-EPO group of mice were comparable with that in the NC group of mice and were significantly lower than that in the HFD-Con group of mice at the end of the experiment (p<0.01, Fig. 1B). Compared with that in the HFD-Con group, the concentrations of fasting serum insulin were significantly reduced by near 50% (p<0.05) although they were significantly higher than that in the NC group of mice (Fig. 1C).

Furthermore, we performed an intraperitoneal glucose tolerance test (IPGTT) in the different groups of mice and found that the dynamic changes in the levels of blood glucose in the HFD-EPO group of mice were similar to that in the NC group and the concentrations of blood glucose in the HFD-EPO group of mice were obviously lower than that in the HFD-Con group of mice following glucose challenge (Fig. 1D). Quantitative analysis revealed that the values of AUC for blood glucose in the HFD-EPO group of mice were similar to that in the NC group and significantly less than that in the HFD-Con group of mice (p<0.01, Fig. 1E). Collectively, these data clearly indicated that treatment with EPO after the establishment of obesity-related T2D significantly reduced the body weights, corrected hyperglycemia and glucose intolerance, and mitigated the HFD-induced hyperinsulinemia in HFD-fed mice.

### The transcription of EPOR mRNA in the liver of mice

**Figure 2:**
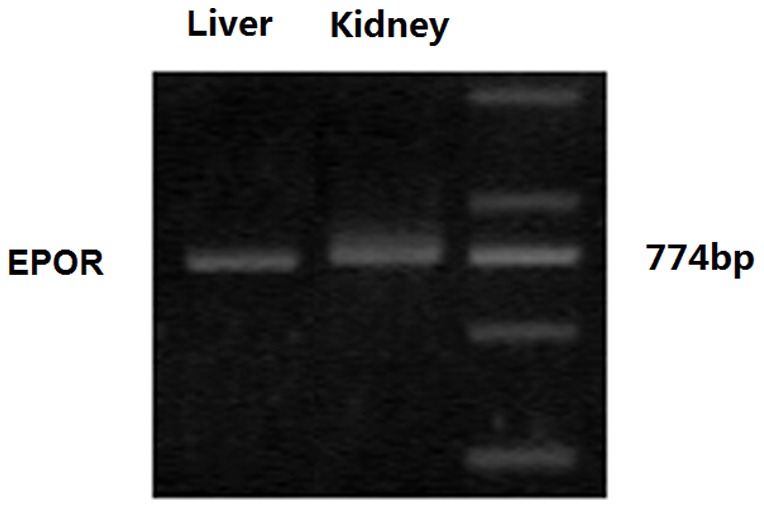
The EPOR mRNA transcription in the livers of mice. Total RNA was extracted from the liver and kidney samples of individual mice and reversely transcribed into cDNA. The EPOR mRNA transcripts were detected by semi-quantitative RT-PCR. Data shown are representative images of agarose gel electrophoresis from three separate experiments.

EPO binds to its receptor, EPOR, which mediates downstream signaling and biological function. To understand the mechanisms underlying the action of EPO, we first tested the levels of EPOR mRNA transcription in the livers of mice by semi-quantitative RT-PCR. We detected the EPOR mRNA transcripts in both the livers and kidneys of mice, suggesting that the EPOR was expressed and mediated its regulatory effect on gluconeogenesis in the livers of mice (Fig. 2).

### EPO enhances the Akt, but not IR and IRS, activation in the livers of mice

**Figure 3:**
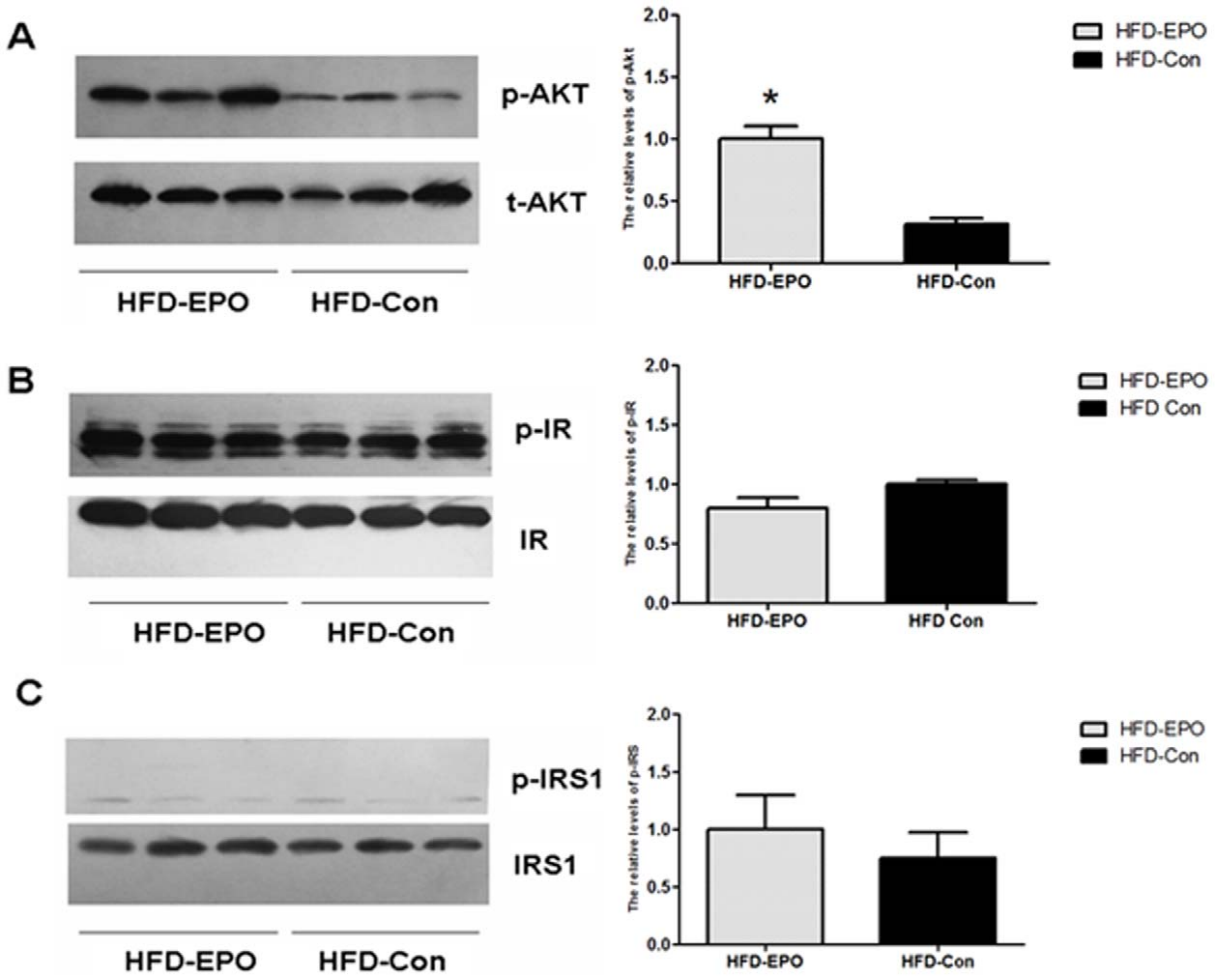
The effect of EPO on the levels of insulin signaling events in the liver. The levels of total Akt, IR and IRS1 and phosphorylated Akt, IR and IRS1 in the livers of individual mice were determined by Western blot assays and the relative levels of Akt, IR and IRS1 phosphorylation were analyzed by densimetric analysis using ImageJ software. Data shown are representative images and expressed as the mean ± SEM of individual groups (n = 6 per group) of mice from three separate experiments. (**A**) Western blot and quantitative analyses of the levels of Akt activation. (**B**) Western blot and quantitative analyses of the levels of IR activation. (**C**) Western blot and quantitative analyses of the levels of IRS1 activation. *P<0.05 vs. the HFD-Con group.

Engagement of EPOR by EPO activates the PI3K/Akt pathway, which participates in the insulin and IR-mediated signaling [31]. To understand the mechanisms underlying the action of EPO in gluconeogenesis, we characterized the levels of total Akt, IR, IRS1 and phosphorylated Akt, IR and IRS1 in the livers of HFD-fed mice by Western blot assays. We found that there were similar levels of Akt, IR and IRS expression in the livers between the HFD-EPO and HFD-Con groups of mice (Fig. 3), suggesting that EPO treatment did not alter the levels of these event expression in the livers of mice. Furthermore, the ratios of the levels of phosphorylated Akt (p-Akt) to total Akt (t-Akt) in the liver tissues were significantly greater in the HFD-EPO group than that in the HFD-Con group of mice (p<0.05, Fig. 3A). However, there was no significant difference in the levels of phosphorylated IR and IRS1 in the livers between the HFD-EPO and HFD-Con groups of mice (Fig. 3B and C). Hence, treatment with EPO enhanced the Akt, but not IS and IRS1, activation in the livers of mice.

### EPO inhibits the PEPCK and G6Pase expression in the liver of mice

**Figure 4:**
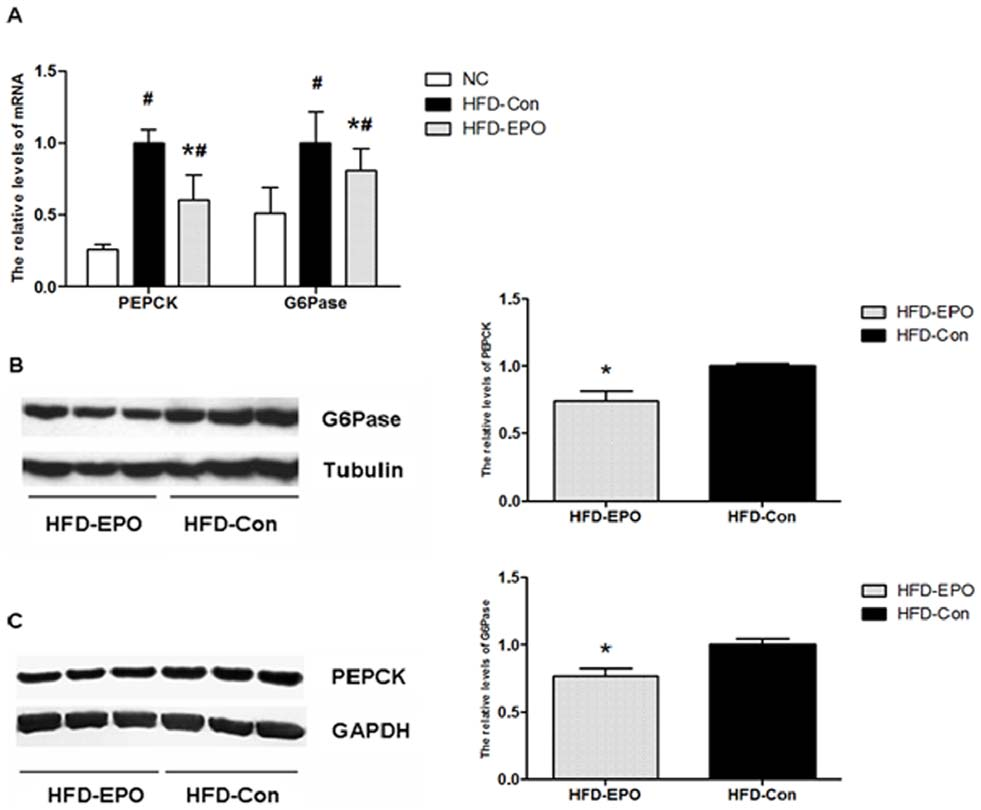
The effect of EPO on the expression of PEPCK and G6Pase in the liver. The relative levels of PEPCK and G6Pase in the livers of individual mice were determined by quantitative RT-PCR and Western blot assays. Data are representative images or expressed as the mean ± SEM of individual group (n = 8 per group) of mice from three separate experiments. (**A**) RT-PCR analysis of the relative levels of PEPCK and G6Pase mRNA transcripts to control GAPDH in the liver. (B) Western blot and quantitative analysis of the relative levels of PEPCK in the liver. (**C**) Western blot and quantitative analysis of the relative levels of G6Pase in the liver. ^#^P<0.05 vs. the NC group, *P<0.05 vs. the HFD-Con group.

The PI3K/Akt activation can regulate the PEPCK and G6Pase expression, which are two rate-limiting enzymes, controlling gluconeogenesis in hepatocytes [13], [14]. Next, we examined whether EPO could modulate the PEPCK and G6Pase expression in the livers of HFD-fed mice by RT-PCR and Western blot assays. We found that the relative levels of PEPCK and G6Pase mRNA transcripts in the livers from the HFD-EPO groups of mice were significantly lower than that from the HFD-Con group although they were higher than that from the NC group of mice (Fig. 4A). Similarly, the relative levels of PEPCK and G6Pase proteins in the livers of HFD-EPO group of mice were significantly reduced, as compared with that in the HFD-Con group of mice (Fig. 4B and C). Thus, EPO treatment significantly inhibited the PEPCK and G6Pase expression in the livers of HFD-fed mice.

### EPO mitigates obesity-related inflammatory signaling in the livers of mice

**Figure 5:**
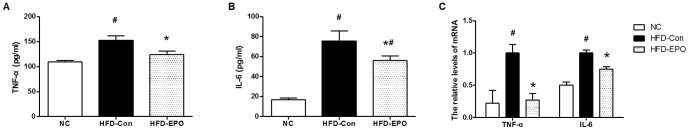
The effect of EPO on inflammatory cytokine expression in mice. At the end of treatment, blood samples were obtained from individual mice and the levels of serum TNF-α and IL-6 were measured by ELISA. In addition, the relative levels of TNF-α and IL-6 mRNA transcripts to control GAPDH were analyzed by quantitative RT-PCR. Data are expressed as the mean ± SEM of the concentrations of serum cytokines or the relative levels of cytokine mRNA transcripts to control GAPDH in each group (n = 8) from three separate experiments. (A) ELISA for the levels of serum TNF-α. (B) ELISA for the levels of serum IL-6. (C) RT-PCR analysis of the relative levels of cytokine mRNA transcripts. ^#^P<0.05 vs. the NC group, *P<0.05 vs. the HFD-Con group.

Chronic inflammation is associated with the development of glucose intolerance and insulin resistance. To further understand the mechanisms underlying the action of EPO, we examined the concentrations of serum inflammatory cytokines by ELISA. In comparison with that in the NC group of mice, we detected significantly higher levels of serum TNF-α and IL-6 in the HFD-Con group of mice, indicating that HFD feeding induced systemic inflammation in mice (Fig. 5A and B). In contrast, the levels of serum TNF-α and IL-6 in the HFD-EPO group of mice were significantly lower than that in the HFD-Con group of mice although the levels of IL-6, but not TNF-α, remained higher than that in the NC group of mice. A similar pattern of the relative levels of TNF-α and IL-6 mRNA transcripts in the livers was detected in the different groups of mice (Fig. 5C). Clearly, these data demonstrated that EPO treatment mitigated the HFD-up-regulated inflammatory cytokine expression in mice.

**Figure 6:**
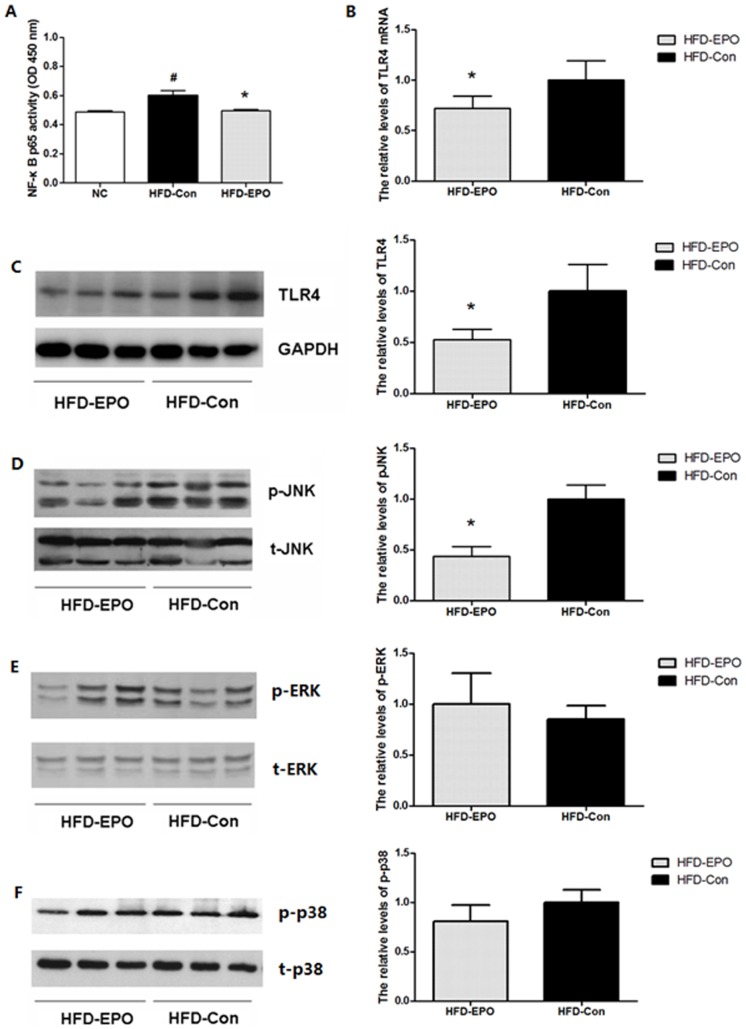
The effect of EPO on the expression of inflammatory signaling events in the liver. The levels of activated NF-*κ*B p65 in the liver were determined by DNA binding-based ELISA (**A**). The relative levels of TLR4 expression were characterized by RT-PCR (**B**) and Western blot assays (**C**). The relative levels of JNK (**D**), ERK (**E**) and p38 MAPK (**F**) phosphorylation in the liver were characterized by Western blot assay and densimetric analysis. Data are representative images or expressed as the mean ± SEM of each group (n = 6) of mice from three separate experiments. ^#^P<0.05 vs. the NC group, *P<0.05 vs. the HFD-Con group.

The NF-κB pathway is a central regulator of inflammation and controls inflammatory cytokine expression. We further investigated whether EPO treatment could alter the NF-κB activation in the liver by a DNA binding-based ELISA assay. We found that the activity of NF-κB p65 binding to the specific DNA in the livers from the HFD-EPO group of mice was similar to that from the NC group of mice and significantly lower than that from the HFD-Con group of mice (p<0.05, Fig. 6A). Accordingly, these data indicated that EPO treatment significantly mitigated the HFD-enhanced NF-κB activation in the livers of mice.

TLR4 is a transmembrane receptor of free fat acid and pathogens, and can activate the NF-κB pathway. We also characterized the levels of TLR4 expression in the livers from the HFD-fed mice by RT-PCR and Western blot assays. We found that the relative levels of TLR4 mRNA transcripts and proteins in the livers from the HFD-EPO group of mice were significantly reduced, as compared with that in the HFD-Con group of mice (p<0.05, Fig. 6B and C). Notably, TLR4 and other inflammatory cytokines can activate the MAPK pathways, including the JNK, ERK and p38 MAPK. We finally tested the JNK, ERK and p38 MAPK expression and activation in the livers of HFD-fed mice by Western blot assays. We found similar levels of total JNK, ERK and p38 MAPK expression in the livers between the HFD-EPO and HFD-Con groups of mice, suggesting that EPO treatment did not modulate their expression in the livers of HFD-fed mice (Fig. 6D–F). However, the relative levels of phosphorylated JNK, but not ERK and p38 MAPK, in the livers from the HFD-EPO group of mice were significantly lower than that from the HFD-Con group of mice. Therefore, treatment with EPO significantly mitigated the HFD-up-regulated TLR4 expression and NF-κB and JNK, but not ERK and p38 MAPK, activation in the livers of HFD-fed mice.

## Discussion

In the present study, we found that EPO treatment significantly reduced the body weights and improved glucose intolerance in the HFD-fed mice. Furthermore, EPO treatment enhanced the PI3K/Akt phosphorylation and mitigated the HFD-induced PEPCK, G6Pase, TNF-α, IL-6 and TLR4 expression, and NF-κB and JNK activation in the livers of mice. These data suggest that EPO-regulated glucose tolerance may be at least partially mediated by enhancing the insulin-related signaling and inhibiting gluconeogenesis and inflammation-related signaling in the livers of HFD-fed mice.

The PI3K/Akt pathway is a crucial regulator of glucose transportation, glycolysis, protein synthesis, lipogenesis, glycogen synthesis and gluconeogenesis [11], [12]. Impairment in the Akt activation is associated with aberrant gluconeogenesis and glucose intolerance while restoration of insulin-induced Akt phosphorylation improves insulin sensitivity and glucose tolerance in the HFD-fed mice [13]. Previous studies have shown that EPO binds to and activates the EPOR, which recruits the p85 subunit of the PI3K through its Src homology 2 (SH2) domain and activates the PI3K and Akt [31], [32]. Subsequently, the activated PI3K/Akt cascade regulates the survival and apoptosis as well as other functions in other non-liver organs [15]–[18]. In the present study, we detected the EPOR mRNA transcription in the liver and found that EPO treatment enhanced the Akt, but not the IR and IRS1, phosphorylation in the livers of HFD-fed mice. These data suggest that EPO may directly activate the PI3K/Akt pathway and overcome the HFD-induced impairment in the insulin-related signaling, leading to the improvement of glucose intolerance in the livers of HFD-fed mice. Our data extended previous findings and indicated that EPO also activated the PI3K/Akt pathway in the livers of HFD-fed mice.

Insulin-related signaling, such as the PI3K/Akt activation, inhibits gluconeogenesis, which is crucial for the maintenance of blood glucose levels and the PEPCK and G6Pase expression, two rate-limiting enzymes that control glucose production [13], [14]. We found that EPO treatment mitigated the HFD-up-regulated PEPCK and G6Pase expression in the livers of HFD-fed mice. Interestingly, we detected significantly lower levels of fasting serum insulin in the HFD-EPO group of mice than that in the HFD-Con group of mice. It is possible that EPO directly activates the PI3K/Akt pathway, which overcomes the HFD-induced impairment in the insulin-related signaling, and inhibits the PEPCK and G6Pase expression, attenuating the HFD-induced gluconeogenesis in the liver and contributing to the improvement of glucose intolerance in HFD-fed mice. Therefore, even after the establishment of insulin resistance, EPO can still activate the PI3K/Akt pathway and inhibit gluconeogenesis, regulating glucose homeostasis.

Chronic low-grade inflammation and pro-inflammatory cytokines, such as TNF-α and IL-6 as well as others, contribute to the development of glucose intolerance and insulin resistance-related metabolic syndrome and T2D [19]–[21], [24]. The TLR4 can activate the NF-κB, JNK, ERK and p38 MAPK pathways, inducing inflammatory cytokine production [22], [33]. We found that EPO treatment mitigated the HFD-up-regulated TLR4 expression, NF-κB and JNK activation and TNF-α and IL-6 production in the livers of HFD-fed mice. Our data were consistent with previous findings that impairment in the TLR4-related NF-κB signaling inhibits the HFD-induced inflammation [20], [34] and activation of EPOR down-regulates TLR4 expression and NF-κB and JNK activation [26], [35]–[37]. Given that TNF-α and IL-6 as well as the NF-κB activation are crucial for the development of glucose intolerance and insulin resistance [22], [24], [38], our data support the notion that inhibition of inflammation-related NF-κB activation may be a promising strategy for the intervention of glucose intolerance. Therefore, our novel findings may provide new insights into the regulation of EPO on inflammation-related glucose intolerance.

The insulin-related PI3K activation can activate the downstream ERK pathway and inflammatory cytokines can also activate the ERK and p38 MAPK pathways. However, we did not observe that EPO treatment significantly altered the ERK and p38 MAPK activation in the livers of HFD-fed mice. The unchanged ERK activation may be complicated by up-regulated PI3K activation in the EPO-treated mice and increased levels of pro-inflammatory cytokine production in the HFD-Con group of mice. The unaltered p38 MAPK activation suggests that the EPOR-related JAK/STAT5 signaling may be insufficient in interfering with the inflammation-related p38 MAPK activation in the livers of HFD-fed mice. We are interested in further investigating the cross-talk between the EPOR-related signaling and pro-inflammatory cytokine-mediated JAK/STAT signaling in hepatocytes.

The mitochondrial oxidative phosphorylation in the liver and skeletal muscles is associated with the development of obesity and insulin resistance [39]. A previous study has shown that EPO treatment enhances muscular fat oxidation, improving insulin sensitivity [7]. In this study, we centered on the effect of EPO on glucose tolerance and gluconeogenesis as well as the possible mechanisms in the livers of HFD-fed mice. We found that EPO treatment significantly reduced the body weights in HFD-fed mice and we speculate that EPO treatment may also enhance fat oxidation in the liver, contributing to the improvement of glucose intolerance in the HFD-fed mice. Now, we are investigating how EPO-related signaling regulates hepatic mitochondrial oxidative phosphorylation and lipid metabolism in the HFD-fed mice.

### Conclusion

Our data indicated that EPO treatment significantly reduced the body weights and the levels of fasting blood glucose and serum insulin, and improved glucose intolerance in the HFD-fed mice. Furthermore, we found that EPO treatment significantly enhanced the PI3K/Akt activation, but inhibited the PEPCK and G6Pase expression in the livers of HFD-fed mice. In addition, EPO treatment mitigated the HFD-induced TLR4 expression, the NF-κB and JNK activation and TNF-α and IL-6 expression in the livers of HFD-fed mice. Collectively, our data suggest that EPO may activate the Akt pathway and inhibit gluconeogensis and inflammation-related signaling in the liver, leading to the improvement of glucose intolerance in the HFD-fed mice. Hence, our findings may provide new insights into the mechanisms by which EPO regulates glucose tolerance and insulin resistance. Given that EPO has been used in humans, EPO or new EPOR agonists may be valuable for the intervention of glucose intolerance-related metabolic syndrome and T2D.
